# A necessary evil? Intra-abdominal hypertension complicating burn patient resuscitation

**DOI:** 10.1186/1752-2897-8-12

**Published:** 2014-08-09

**Authors:** Paul B McBeth, Kim Sass, Duncan Nickerson, Chad G Ball, Andrew W Kirkpatrick

**Affiliations:** 1 Departments of Surgery, Foothills Medical Centre, University of Calgary, Calgary, Alberta, Canada; 2 Critical Care Medicine, Foothills Medical Centre, University of Calgary, Calgary, Alberta, Canada; 3 Regional Trauma Program, Foothills Medical Centre, University of Calgary, 1403 – 29th Street N.W., Calgary, Alberta, Canada

**Keywords:** Abdominal compartment syndrome, Intra-abdominal hypertension, Burn, Fluid resuscitation, Critical care

## Abstract

**Objective:**

Severe burns are devastating injuries that result in considerable systemic inflammation and often require resuscitation with large volumes of fluid. The result of massive resuscitation is often raised intra-abdominal pressures leading to Intra-abdominal hypertension (IAH) and the secondary abdominal compartment syndrome. The objective of this study is to conduct (1) a 10 year retrospective study to investigate epidemiological factors contributing to burn injuries in Alberta, (2) to characterize fluid management and incidence of IAH and ACS and (3) to review fluid resuscitation with a goal to identify optimal strategies for fluid resuscitation.

**Design:**

A comprehensive 10-year retrospective review of burn injuries from 1999.

**Outcome Measures:**

Age, sex, date, mechanism of injury, location of incident, on scene vitals and GCS, type of transport to hospital and routing, ISS, presenting vitals and GCS, diagnoses, procedures, complications, hospital LOS, ICU LOS, and events surrounding the injury.

**Results:**

One hundred and seventy five patients (79.4% M, 20.6% F) were identified as having traumatic burn injuries with a mean ISS score of 21.8 (±8.3). The mean age was 41.6 (±17.5) (range 14-94) years. Nearly half (49.7%) of patients suffered their injuries at home, 17.7% were related to industrial incidents and 14.3% were MVC related. One hundred and ten patients required ICU admission. ICU LOS 18.5 (±8.8) days. Hospital LOS 38.0 (±37.8) days. The mean extent of burn injury was 31.4 (±20.9) % TBSA. Nearly half of the patients suffered inhalational injuries (mild 12.5%, moderate 13.7%, severe 9.1%). Thirty-nine (22.2%) of patients died from their injuries. Routine IAP monitoring began in September, 2005 with 15 of 28 patients having at least two IAP measurements. The mean IAP was 16.5 (±5.7) cm H_2_O (range: 1-40) with an average of 58 (±97) IAP measurements per patient. Those patients with IAP monitoring had an average TBSA of 35.0 (±16.0)%, ISS of 47.5 (±7.5). The mean 48 hr fluid balance was 25.6 (±11.1)L exceeding predicted Parkland formula estimates by 86 (±32)%.

**Conclusions:**

Further evaluation of IAP monitoring is needed to further characterize IAP and fluid resuscitation in patients with burn injuries.

## Introduction

Severe burns are devastating injuries resulting in a considerable systemic inflammatory response often requiring resuscitation with large volumes of fluid [1]. The end result of massive resuscitation is often elevated intra-abdominal pressures (IAP). The influence of raised IAP known as intra-abdominal hypertension (IAH) is being recognized has having effects on all aspects critically ill patient physiology [2,3]. The most extreme manifestation of IAH, is new onset organ failure in the setting of an IAP greater than 20 mmHg defined as the abdominal compartment syndrome (ACS) [4]. While IAH and ACS were classically described after damage control surgery from trauma and patients undergoing massive fluid resuscitation [5-8], these entities have also been noted in many different clinical settings that are unified by the simple condition of being critically ill and thus requiring massive fluid resuscitation. Recent literature suggests resuscitation induced or secondary ACS without abdominal injury is relatively common with an associated increased mortality rate [9,10]. Ivy has identified 250cc/kg of volume administration within the first 24 hours as a risk factor for ACS [11]. IAH and ACS are both well-described entities associated with patients having severe burn injuries. The extent of burn injury appears to be directly related to development of ACS with patients having >70% TBSA burn almost inevitably will get ACS [12]. Management of ACS with decompressive laparotomies is associated with significant morbility and mortality ranging from 50% to 100% [13]. However, novel resuscitation strategies in burn patients to avoid IAH/ACS are evolving. Recent evidence supports the use of hypertontic sodium chloride solution and colloids enabling less overall fluid volume resuscitation. Despite efforts to minimize fluid administration many patients end up grossly fluid overloaded leading to IAH and ACS [14-16].

The objective of this study is to conduct (1) a 10 year retrospective study to investigate epidemiological factors contributing to burn injuries in Alberta, Canada captured within the Southern Alberta Trauma Registry (SATR), (2) to characterize fluid management and incidence of IAH and ACS and (3) to identify optimal strategies for fluid resuscitation. We hypothesize the incidence of severe burn injuries are not uncommon and current resuscitation strategies are likely variable.

## Methods

Patients with an Injury Severity Score (ISS) greater than 12 resulting from traumatic burn accidents were identified using the SATR at the Foothills Medical Centre (FMC) in Calgary, Alberta, Canada. The Foothills Medical Centre is an adult tertiary care trauma referral center responsible for all major trauma care in Southern Alberta and Eastern British Columbia. It serves as a referral center for a population of approximately 1.5 million people. The study cohort included all patients admitted between May 1, 1999 and April 30, 2009 with burn or inhalation injuries. The University of Calgary Institutional Review Board approved the study prior to its initiation. Patients with IAP monitoring had a standard intravesical catheter inserted as per our standard institutional practice for critically ill patients at risk of IAH. Our institution has adopted practice guidelines as outlined by the World Society of the Abdominal Compartment Syndrome (WSACS) for IAP monitoring. Frequent IAP monitoring is currently an expected standard for patients with a diagnosis of sepsis, multisystem trauma, or those requiring vigorous fluid resuscitation. IAP monitoring employed either a standard 2-way catheter or the irrigation port of the 3-way catheter as a conduit connected to a pressure transducer. The pressure transducer was placed in-line with the iliac crest at the mid-axillary line and interfaced with a multichannel bed-side monitor (GE Marquette–Solar 7000 Patient Monitor, GE Healthcare Technologies, Waukesha, WI). Subjects were positioned supine for each measurement cycle. Intermittent measurements were made after instilling 20 mL normal saline into the bladder and clamping the tubing distally. Each IAP measurement was obtained at the first end-expiratory pause occurring 60 seconds after repositioning, or when the patient was settled (if the patient was agitated) to allow for reduced transient pressure artifact. IAP and other physiologic parameters were stored in an electronic database for all patients admitted to the ICU.

For each patient included in the study, the following data were captured from the SATR: age, sex, ISS, on scene and presenting vital signs, GCS score, injury, mechanism of injury, location of incident, type of transport to hospital and routing, diagnoses, procedures performed, hospital and ICU length of stay, events surrounding the injury, time of year, and time of day. Thereafter patients identified in the SATR were cross-referenced to the Critical Care Physiologic Database (CCPD) used to identify patients admitted to the ICU with measurement of physiologic parameters related to fluid resuscitation. These parameters include: duration in ICU and on the ventilator, fluid administration, IAP measurements, critical illness assessment scores (SOFA, MPM, APACHE, TISS, CHP), GCS, vital signs and ICU disposition. Finally, a formal chart review was completed in any case with missing patient registry data in either database.

Analysis was performed using Stata version 8.0 (Stata Corp, College Station, TX). The mean, median, and range were calculated using standard methodology. Data is reported as means when normally distributed, and medians when non-normally distributed.

## Results

**Figure 1:**
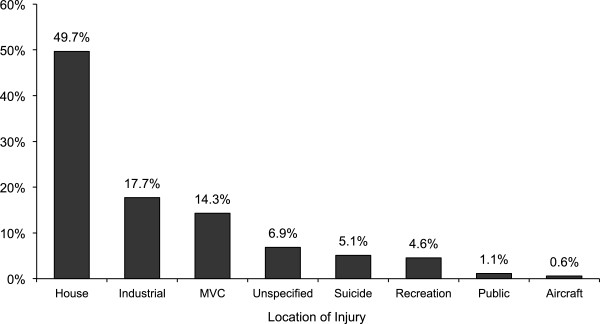
Location of accident.

**Figure 2:**
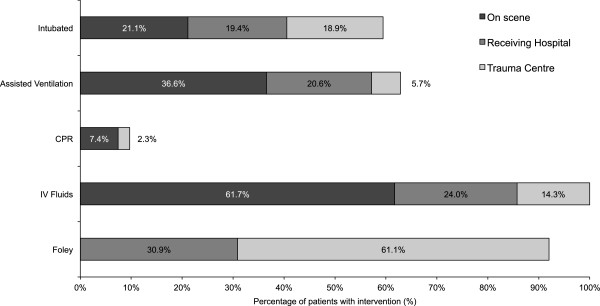
Interventions for burn patient resuscitation.

During the 10-year study period 8847 injured adult patients were treated at FMC with an ISS ≥ 12. A total of 175 (1.9%) patients were admitted with burn or inhalation injuries. The majority were male (79.4%) with a mean age of 40.5 ± 15.9 years; 20.6% Female, mean age 45.6 ± 22.2 years; overall mean age 41.6 ± 17.5 years (range: 14 - 94 years). The mean ISS was 21.8 ± 8.3. One hundred and ten (62.8%) patient’s required ICU support and thirty-nine (22.3%) patients died. The majority of injuries were sustained during house fires (49.7%) and industrial accidents (17.7%) (Figure 1). The mechanisms of injury included: enclosed space fires: 101 (57.7%), open fires: 39 (22.3%), explosions: 24 (13.7%), and electrical: 11 (6.3%). Most injuries occurred during the summer (Summer: 62 (35.4%), Spring: 42 (24.0%), Winter: 37 (21.2%), Fall: 34 (19.4%)).Resuscitation of patients on scene and at the initial receiving hospital was variable. On scene interventions included assisted ventilation: 64 (36.5%), intubation: 37 (21.1%), CPR: 13 (7.4%), and administration of IV fluids: 108 (61.7%) (Figure 2). One (0.5%) patient required a cricothyroidotomy. The majority (82.3%) of patients were transported by ground ambulance. Two percent of patients were transported directly from the scene via helicopter and 9.7% utilized combined ground ambulance and aircraft. The remaining 8.0% of patients were transported to hospital in a private vehicle. The mean transport time (i.e. from the time of injury to arrival at the FMC) was 3.1 ± 2.6 hours.

**Figure 3:**
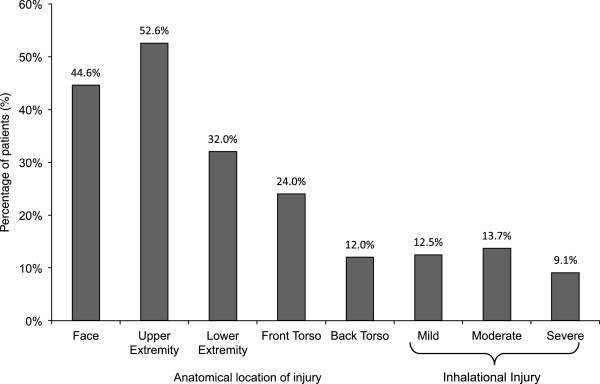
Burn injury distribution.

**Figure 4:**
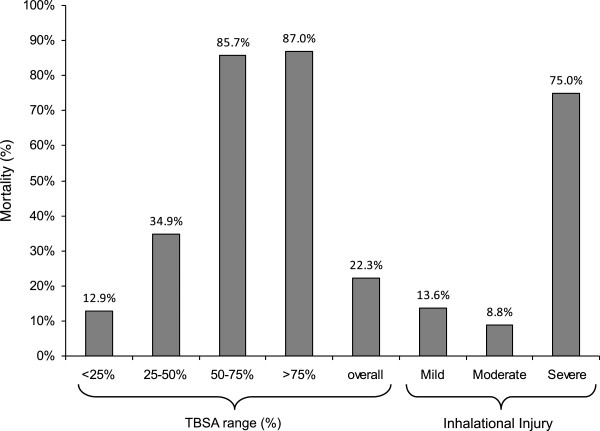
Burn injury associated mortality.

The mean GCS of patients arriving at FMC was 11.9 ± 4.7. Seventy-one (40.6%) patients arrived already intubated. An additional six patients required intubation as part of ongoing resuscitation. A total of 17 (9.7%) patients required CPR. Of those requiring CPR, 10 (5.7%) patients were pronounced dead in the trauma bay, seven (4.0%) patients went to the ICU and of those only two (1.1%) patients survived. One hundred and fifty (85.7%) patients arrived with administration of IV fluids and only 68 (39.1%) patients had a foley catheter placed.An estimate of the Total Body Surface Area (TBSA) burn was completed by the on-call plastic surgeon. The average TBSA burn was 31.4 ± 20.9% (range: 5-95%). The majority of burns were to the face (44.6%) and upper extremity (52.6%) including hands (Figure 3). Seventy-two (41.1%) patients suffered inhalation injuries (Mild: 22 (12.5%), Moderate: 24 (13.7%), Severe: 16 (9.1%)). Mortality rates increased with percentage TBSA and degree of inhalation injury (Figure 4). Intra-abdominal pressure increased with the severity of inhalation injury: Mild (IAP: 15.2 ± 4.9 mmHg), Moderate (IAP: 16.1 ± 5.1 mmHg), and Severe (IAP: 17.0 ± 4.3 mmHg). Other associated injuries included: extremity (13.1%), abdominal (4.6%), facial injuries (2.9%), head (1.7%), spinal (1.1%), and hypothermia (1.1%).

One hundred and ten patients (62.8%) went to the ICU initially however only 93 (53.1%) stayed greater than 24 hrs. Eighty-eight (50.2%) patients required ventilatory support and 19 (10.9%) patients required vasopressive support. Mean number of days on the ventilator was 13.7 ± 11.6 days (range 2-81). The MPM and ISS was 22.1 ± 23.8 (range 2-81) and 45.9 ± 8.3 (range 22-60) respectively. One hundred and forty-eight (84.6%) patients required a surgical intervention. The majority of these procedures were wound debridements (46.9%) followed by 23 (13.1%) fasciotomies, and 13 (7.4%) amputations. Fourteen (8.0%) patients required laparotomies of which three (1.7%) were related to decompression for ACS.

**Figure 5:**
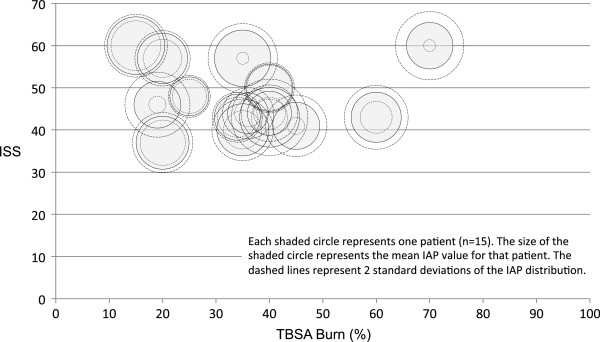
IAP distribution associated with ISS and TBSA burn.

Routine institutional IAP monitoring began in September 2005. Since then 53 (30.2%) burn patients were identified using the SATR. Twenty-eight required ICU admission. Of those admitted to the ICU only 15 (53.6%) patients had at least two IAP measurements with three patients having only one measurement. The mean number of IAP measurements per person was 58 ± 97 (range: 2-363) (Figure 5). The mean IAP across all patients was 16.5 ± 5.7 mmHg (range 1-40) with 12 of 15 patients having severe IAH with pressures greater than 20 mmHg. *Of the 15 patients with IAP monitoring the mean time from injury to the first IAP measurement was 2.3 *±* 2.1 days. Patients with IAP > 20 the mean peak in IAP was 3.6 ± 2.7 days after the time of injury.* The percent TBSA and ISS was 35.0 ± 16.0 % (range 11-70) and 47.5 ± 7.5 (range 37-60) respectively. *Seven of those patients have significant blood loss (estimated > 1L) associated with other injuries (hemothorax, extremity orthopedic and vascular injuries, and intra-abdominal injuries) unrelated to the patients burn injury. These patients received resuscitation aimed at both blood loss replacement from traumatic injury and for management of their burns.* The mean ICU LOS was 18.5 ± 8.8 days (range 6-34). *Of the 12 patients with IAP > 20 only 5 patients developed new onset organ dysfunction. Each patient had a greater than 50% rise in creatine. Three patients went on to develop anuric AKI requiring CRRT support.* The mean 48 hr fluid balance: 25.6 ± 11.1 L (range 11.3-51). The initial 24 hr fluid resuscitation exceeded the predicted Parkland formula estimates by 86 ± 32%. *Of the 12 patients with IAP > 20, 8 patients received active treatment in an attempt to reduce IAP. These treatments included: gastrointestinal decompression with nasogastric suctioning, increased sedation goals, and fluid removal. No patients in this study received a paracentesis for fluid removal despite our recent interest in this method as a means of avoiding formal decompressive laparotopmy. The primary trigger for decompressive laparotomy appeared to be difficultly with mechanical ventilation. Three patients receiving decompressive laparotomies had peak airway pressure greater than 35 mmHg with low tidal volume ventilation. On average the decompressive laparotomy took place 4.2 *± *2.3 days after injury.*

Thirty-nine (22.2%) patients died as a result of their injury. The majority were related to overwhelming sepsis. The mean LOS in hospital was 38.0 ± 37.8 days (range: 0-215 days). The majority of patients were either transferred to the Burn (57.1%) or Trauma (19.7%) Units.

## Discussion

Severe burns are often devastating injuries resulting in a profound systemic inflammation response requiring large volume fluid resuscitation. The associated third spacing of fluids can result in swelling of almost any body compartment, often resulting in considerable morbidity and mortality. This is especially true as it relates to the abdominal compartment. Massive bowel swelling and peritoneal ascites raises IAP leading to IAH and ACS.

IAP monitoring is important in critically ill burn patients because of the potentially fatal consequences of IAH and ACS. Physiological changes occur with increases in IAP that affect nearly every organ system. IAH and ACS are well described in the surgical literature and associated with primary intra-abdominal pathology and in patients undergoing large-volume resuscitation. A recent European study demonstrated that 4.2% of patients (medical and surgical) admitted to ICU had ACS and 32.1% had IAH [17] at the time of admission. The same group found mortality rates were higher in patients with ACS and that fluid resuscitation was an independent risk factor for the development of IAH. Furthermore, Fuchs and colleagues demonstrated that patients undergoing large volume resuscitation (5 L or more net positive fluid balance in 24 hours) possessed incidence rates of 85% and 25% for IAH and ACS respectively [18]. Patients with severe burn injuries greater than 60%, associated inhalational injuries, delayed resuscitation, and intra-abdominal injuries are at the highest risk of developing IAH and ACS. Management strategies are targeted at sedation, gastrointestinal decompression, escharotomies and drainage of ascites [19]. Decompressive laparotomies should be performed in patients with ACS when non-surgical methods fail [20].The associations from our data analysis were generally not robust enough for formal categorization of IAH. The observed variability may be due to the small data set and modest IAP monitoring in patients with server burns. Of the 28 patients admitted to the ICU only 15 had at least two IAP measurements, despite international and institutional guidelines suggesting IAP monitoring in patients with burns. Of those with IAP monitoring we observed trends towards higher mortality among those with higher mean IAP measurements. There did not appear to be an association of IAP with ISS or TBSA (Figure 5). Despite this, patients with severe burn injuries clearly received volume resuscitation exceeding traditional Parkland guidelines by 86%. Severe inhalation injuries appear to be related to the degree of fluid resuscitation and the development of abdominal compartment syndrome.

This study had a number of limitations. First, as a retrospective study the possibility of bias could not be eliminated. Second, the study was limited by the documented data fields collected in the SATR and CCPD. Details outlining the events preceding the injury were not well described and standard monitoring of IAP was found to be uncommon.

Despite these limitations, our study characterized burn injury patterns, current resuscitation methods, and the prevalence of IAP monitoring and associated IAH and ACS. Most of the injuries observed occurred in young males and resulted from residential or industrial fires. Burn injuries to the face and upper extremity were most common. Early aggressive fluid resuscitation was demonstrated in the majority of patients while IAP was not commonly measured. Those with IAP monitoring the majority had IAP’s >20 mmHg. Traditional guidelines for initial fluid resuscitation such as the Parkland Formula were often grossly exceeded, putting patients at risk of developing IAH and ACS.

## Conclusions

Severe burns result in profound systemic inflammation response often requiring large volume fluid resuscitation exceeding traditional fluid requirement estimates. Elevated IAP resulting from aggressive fluid resuscitation mandates IAP monitoring in patients with severe burn injuries. Novel resuscitation strategies to avoid or minimize IAH/ACS are needed and require further study.

## Competing interests

The authors declare that there is no actual or potential conflict of interest in relation to this article.

## Authors’ contributions

PBM and AWK drafted the manuscript. PBM, KS, DN and CGB contributed to acquisition of data, analysis and interpretation of data. AWK, CGB and DN participated in conception, design and coordination, and supervised the whole study. All authors read and approved the final manuscript.
